# The Hypoxia-Associated Localization of Chemotaxis Protein CheZ in *Azorhizorbium caulinodans*

**DOI:** 10.3389/fmicb.2021.731419

**Published:** 2021-10-15

**Authors:** Xiaolin Liu, Yanan Liu, Yixuan Wang, Dandan Wang, Kevin Scot Johnson, Zhihong Xie

**Affiliations:** ^1^Key Laboratory of Coastal Environmental Processes and Ecological Remediation, Yantai Institute of Coastal Zone Research, Chinese Academy of Sciences, Yantai, China; ^2^College of Resources and Environment, University of Chinese Academy of Sciences, Beijing, China; ^3^National Engineering Laboratory for Efficient Utilization of Soil and Fertilizer Resources, College of Resources and Environment of Shandong Agricultural University, Taian, China; ^4^Department of Microbiology and Environmental Toxicology, University of California, Santa Cruz, Santa Cruz, CA, United States

**Keywords:** chemotaxis, hypoxia, localization, CheZ, *Azorhizobium caulinodans*

## Abstract

Spatial organization of chemotactic proteins is important for cooperative response to external stimuli. However, factors affecting the localization dynamics of chemotaxis proteins are less studied. According to some reports, the polar localization of chemotaxis system I is induced by hypoxia and starvation in *Vibrio cholerae*. However, in *V. cholerae*, the chemotaxis system I is not involved in flagellum-mediated chemotaxis, and it may play other alternative cellular functions. In this study, we found that the polar localization of CheZ, a phosphatase regulating chemotactic movement in *Azorhizobium caulinodans* ORS571, can also be affected by hypoxia and cellular energy-status. The conserved phosphatase active site D165 and the C-terminus of CheZ are essential for the energy-related localization, indicating a cross link between hypoxia-related localization changes and phosphatase activity of CheZ. Furthermore, three of five Aer-like chemoreceptors containing PAS domains participate in the cellular localization of CheZ. In contrast to carbon starvation, free-living nitrogen fixation can alleviate the role of nitrogen limitation and hypoxia on polar localization of CheZ. These results showed that the localization changes induced by hypoxia might be a strategy for bacteria to adapt to complex environment.

## Introduction

*Azorhizobium caulinodans* ORS571 is an alpha-*Proteobacterium* that can fix atmospheric nitrogen in free-living conditions or inside nodules formed on root or stem of the tropical legume *Sesbania rostrata* (Dreyfus and Dommergues, 1981; Dreyfus et al., 1988). Chemotaxis and motility provide a fitness advantage for *A. caulinodans* ORS571 during host colonization and allows for adaptation to harsh environment (Jiang et al., 2016a; Liu et al., 2017, 2018a,b, 2019; Sun et al., 2019).

The chemotaxis signaling pathway has been well studied in *Escherichia coli* (Wadhams and Armitage, 2004). Transmembrane methyl-accepting chemotaxis proteins (MCPs) are responsible for sensing external stimuli. The presence of repellents or absence of attractants can induce conformational changes in MCPs resulting in increased activity of the histidine kinase CheA. CheA phosphorylates the response regulator CheY, and then CheY-P diffuses in the cytoplasm and ultimately interacts with FliM, a flagellar motor protein, changing the direction of movement. The phosphatase CheZ promotes the dephosphorylation of CheY-P and terminates the signal transduction. The methyltransferase CheR and the methylesterase CheB interacting with CheA and MCPs are involved in chemotaxis adaptation (Wadhams and Armitage, 2004; Sourjik and Armitage, 2010). In addition to transmembrane MCPs, cytoplasmic chemoreceptors containing PAS domains, such as Aer in *Escherichia coli* and AerC in *Azospirillum brasilense*, are involved in the orientation movement along oxygen and redox gradients (Alexandre et al., 2004). To amplify small signals and improve positive cooperativity, two chemoreceptors, one CheA dimer, and two adaptor proteins CheW form core units, and these units cluster in highly ordered supramolecular arrays at subcellular poles (Hazelbauer et al., 2008; Pinas et al., 2016). The *cheA* gene contains two tandem translation starts and separately encodes CheA-long and CheA-short with a common C-terminus in *E. coli* (Wang and Matsumura, 1996; O'connor et al., 2009). CheZ localizes to cell poles by binding with CheA-short, which lacks the first 97 residues of full-length CheA, termed as CheA-long (Wang and Matsumura, 1996; Sourjik and Berg, 2000; Cantwell et al., 2003). The localization pattern of chemotaxis proteins at the cellular poles was proposed to result from the high membrane curvature of cells poles (Draper and Liphardt, 2017) or to the presence of specific lipids or proteins at cell poles (Laloux and Jacobs-Wagner, 2014; Saaki et al., 2018). In addition, the polar location could be explained with nucleoid occlusion and “stochastic self-assembly” model (Jones and Armitage, 2015; Neeli-Venkata et al., 2016). The physiological significance of protein polar localization may lie in that both daughter cells can inherit a chemosensory cluster, which is critical for survival in complex environment (Jones and Armitage, 2015).

The genome of *A. caulinodans* ORS571 contains 43 genes encoding chemoreceptors, and seven genes encoding the sole chemotaxis pathway-related proteins, including one core chemotaxis cluster (*cheA*, *cheY2*, *cheW*, *cheB*, and *cheR*) and two orphan genes, *cheY1* and *cheZ* (Jiang et al., 2016b). Only two chemotactic proteins, IcpB and CheZ, have been marked with fluorescent tags to determine their cellular location (Jiang et al., 2016a). IcpB, which has one PAS domain, is critical for aerotaxis and chemotaxis (Jiang et al., 2016a). In addition, IcpB, which is similar to soluble receptor McpS in *Pseudomonas aeruginosa* and McpY in *Sinorhizobium meliloti*, located to cell poles with the help of CheA (Bardy and Maddock, 2005; Meier and Scharf, 2009; Jiang et al., 2016a). The CheZ proteins, in alpha-*Proteobacteria*, are encoded by an orphan gene adjacent to an orphan *cheY* (Liu et al., 2018b). Disruption of *cheZ* in *A. caulinodans* abolishes the chemotactic response to attractants and causes an increase in exopolysaccharide production (Liu et al., 2018b). Recently, we have determined the localization pattern of CheZ at cell poles and identified a novel motif that is involved in the regulation of localization (Liu et al., 2020).

Hypoxia and carbon starvation have been shown in *V. cholerae* to regulate the localization of chemotaxis-like proteins, which are not involved in flagella-mediated motility (Hiremath et al., 2015; Ringgaard et al., 2015). However, the relationship between hypoxia and the localization of chemotaxis proteins has not been studied in bacteria. Interestingly, under nitrogen limiting environment, nitrogen-fixing rhizobacteria, such as *A. caulinodans* and *A. brasilense*, move toward microaerobic environments to fix nitrogen (Greer-Phillips et al., 2004; Xie et al., 2010; Jiang et al., 2016a) due to the activity of nitrogenase inhibited by high concentration of oxygen (Desnoues et al., 2003). Accordingly, we sought to examine the role of hypoxia in the localization of CheZ in *A. caulinodans* and found that three soluble chemoreceptors are involved in the process. Furthermore, nitrogen limiting conditions are also involved in the polar localization of CheZ. These results indicated that the fluctuation of subcellular localization of chemotaxis proteins might be an important strategy for bacteria to respond to environmental stimulus.

## Materials and Methods

### Bacterial Strains and Growth Conditions

All strains and plasmids used in this study are listed in Table 1. *Escherichia coli* strains were grown in LB media at 37°C. *Azorhizobium caulinodans* ORS571 and derivatives were cultured with tryptone-yeast extract (TY) medium or L3 minimal medium at 37°C. Carbon or nitrogen sources in L3 medium can be removed as needed, which are designated as L3-C+N or L3+C-N. To create the environment with different oxygen concentrations, first, the air in 20ml anaerobic tubes was replaced by the oxygen-free gas N2 for hypoxia environment or gas mixture of N_2_/O_2_ for hyperxia environment. Then 5ml overnight cell liquid culture was injected into the anerobic tubes and was shaken for 30s before putting into incubator with standing or shaking. The final concentrations of antibiotics used in this work are 100μg m^−1^ of ampicillin, 50μg m^−1^ of gentamicin and kanamycin, and 25μg m^−1^ of nalidixic acid.

**Table 1:** Bacterial strains and plasmids used in this study.

Strain or plasmid	Relevant characteristics^a^	Source or reference
Strains		
*E. coli*		
DH5α	F-*supE44 AlacU169 (ɸ80 lacZΔM15) hsdR17 recA1 endA1 gyrA96 thi-1 relA1*	Transgen
*Azorhizobium caulinodans*		
ORS571	Wild-type strain, Amp^R^, Nal^R^	Dreyfus et al., 1988
*ΔcheZ*	ORS571 derivative; *cheZ* deleted mutant, Amp^R^, Nal^R^, Gm^R^	Liu et al., 2018b
*Δ0573*	ORS571 derivative, azc_0573 deleted mutant, Amp^R^, Nal^R^	This study
*Δ1026*	ORS571 derivative, azc_1026 deleted mutant, Amp^R^, Nal^R^, Gm^R^	This study
*Δ1546*	ORS571 derivative, azc_1546 deleted mutant, Amp^R^, Nal^R^	This study
*Δ3153*	ORS571 derivative, azc_3153 deleted mutant, Amp^R^, Nal^R^, Gm^R^	This study
*Δ3718*	ORS571 derivative, azc_3718 deleted mutant, Amp^R^, Nal^R^	Jiang et al., 2016a
Plasmids		
pRK2013	Helper plasmid, ColE1 replicon; Tra+Km^R^	Ditta et al., 1980
pCM351	Allelic exchange vector; Gm^R^, Tc^R^	Marx and Lidstrom, 2002
pK18*mobsacB*	Suicide vector for gene disruption; lacZ *mob sacB* Km^R^	Schäfer et al., 1994
pBBR2	Broad host range plasmid, Km^R^	Kovach et al., 1995
pBBR2CheZ-GFP	pBBR-1-MCS-2 with *cheZ* fused with egfp, Km^R^	Liu et al., 2020
pBBRR2CheZN70	pBBR-1-MCS-2 with *cheZ* remaining 210bp at the N-terminus fused with *egfp*; Km^R^	Liu et al., 2020
pBBRR2CheZD165A	pBBR-1-MCS-2 with *cheZ* with a D165A substitution fused with *egfp*; Km^R^	Liu et al., 2020
pBBRCheZQ169A	pBBR-1-MCS-2 with *cheZ* with a Q169A substitution fused with *egfp*; Km^R^	Liu et al., 2020

a Amp^R^, ampicillin resistance; Gm^R^, gentamycin resistance; Km^R^, kanamycin resistance; Nal^R^, nalidixic acid; and Tc^R^, tetracycline.

### Construction of Soluble Chemoreceptor Mutants

Two methods were used to construct deleted mutants. The Δ*1026* and Δ*3153* were constructed by pK18*mobsacB*, a suicide vector, and the Δ*0573* and Δ*1546* were constructed by pCM351, an allelic exchange vector. To construct plasmids for *azc_1026* and *azc_3153* gene deletion, upstream and downstream fragments of them were amplified with primers listed in Table 2. The 3′ end of upstream fragment and the 5′ end of downstream fragment were amplified with same restriction enzyme (EcoRI). After being digested with three restriction enzymes, the two amplicons were integrated into pK18*mobsacB* to construct a pK18*mobsacB*::up-down plasmid. The resulting plasmid was introduced into *A. caulinodans* ORS571 by triparental conjugation with the help of pRK2013, a helper plasmid. The *azc_1026* and *azc_3153* deletion mutants derived from wild-type strain were designated Δ*1026* and Δ*3153*.

**Table 2:** PCR primers used in this study.

Primers	Sequences (5′-3′)^a^	Purpose for construction
1026up-F	CGGGGTACCGGACGGTGCAGGAGGAGGC	*Δ1026* construction
1026up-R	CGGCATATGGCCTCAATGATTGCTCAC	*Δ1026* construction
1026down-F	CGACGCGTTCCGCCCCTGTCGACAATG	*Δ1026* construction
1026down-R	CGGAGCTCTGGTGAGCTGGATGATGG	*Δ1026* construction
1546up-F	CGCGGATCCTGAGCCATTACGACCCCATC	*Δ1546* construction
1546up-R	CGGAATTCGGAGCCCCCCTTTTTCTCG	*Δ1546* construction
1546down-F	CGGAATTCTCCGGACCGCCGCGCG	*Δ1546* construction
1546down-R	GCTCTAGACACGATGCCTTCAACCTC	*Δ1546* construction
3153up-F	CGGGGTACCGCCGCAAACCCGAAAATCCG	*Δ3153* construction
3153up-R	CGGCATATGGCCGGGGCTTGATCTTCG	*Δ3153* construction
3153down-F	CGACGCGTGGCGGCCCTGCCCGGCA	*Δ3153* construction
3153down-R	CGGAGCTCTGGTTTCGTCGGCCTTGGAG	*Δ3153* construction

a Engineered restriction sites are underlined.

To construct *azc_0573* and *azc_1546* deletion mutants, the upstream and downstream fragments of them were amplified. These amplicons of upstream fragments were firstly digested and introduced into pCM351 to construct a pCM351::up plasmid. Then the downstream fragments of genes were digested and introduced into the pCM351::up plasmid. The resulting plasmids, pCM351::up-down, were integrated into wild-type strain through triparental conjugation for allelic exchange. Homologous recombinants lacking *azc_0573* or *azc_1546* were selected and termed as Δ*0573* and Δ*1546*.

### Microscopy and Data Analysis

*Azorhizobium caulinodans* ORS571 cells were grown to stationary phase (24h), then the effect of standing time or the role of the hypoxia and electron transport chain inhibitors on localization on the cellular localization of CheZ were observed. To immobile cells, 1% agarose pads were constructed as previously described (Meier and Scharf, 2009). The Olympus BX53 system fluorescence microscope with Olympus DP73 digital camera was used to take photos with a 100×objective. The cellSensDimernsion 1.7 imaging software (Olympus Inc.) was used to capture clear images. The number of cells showed diffuse or polar localization of CheZ was recorded using manual count.

### Bioinformatics Analysis

Sequences of intracellular chemoreceptors in *A. caulinodans* ORS571, Aer in *E. coli*, and AerC in *A. barasilense* were collected from Mist 2 database (Ulrich and Zhulin, 2010).^1^ Domain analysis of proteins was performed using SMART program (Letunic and Bork, 2018). The protein sequences were aligned by MAFFT program (Madeira et al., 2019) and built a maximum likelihood tree using MEGA (Kumar et al., 2018).

### Statistical Analysis

All results in this study were subjected to ANOVA. Tukey’s test was used for multiple comparisons, and student *t*-test (*p*<0.01 and *p*<0.05) was used for significant differences between conditions. All tests were performed using SPSS version 20.0 software (IBM Corp., Armonk, New York).

## Results

### Hypoxia Regulates the Polar Localization of CheZ

**Figure 1:**
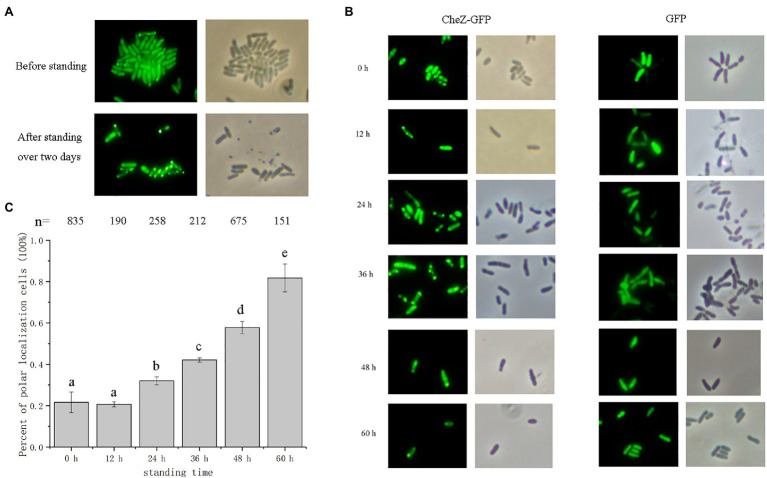
Localization of CheZ-GFP with cell standing time. **(A)** Representative images of overnight culturing *Azospirillum caulinodans* cells with CheZ-GFP before standing or after standing over 2days. **(B)** Representative images of cells with CheZ-GFP or GFP-only with standing time from 0 to 60h. **(C)** Quantification of cell ratios with CheZ polar localization. Values in **(C)** are means and SDs from three independent experiments. The same letter above the bars indicates no significant difference at *p*<0.05, one-way ANOVA with Tukey’s multiple-comparison test.

We previously reported that CheZ in *A. caulinodans* can locate to cell poles and the localization pattern of CheZ is diverse, including monopolar, bipolar, and diffuse (Liu et al., 2020). Among them, diffuse localization of CheZ is the most common, observed in about 60% of cells (Liu et al., 2020). Unexpectedly, we observed that the ratio of cells with polar CheZ localization becomes very high after standing over 2days (Figure 1A). To get a clear conclusion, we quantified them and sought to determine the factors causing higher polar localization of CheZ.

First, we performed a time-course assay of CheZ localization. Cells with CheZ-GFP fusion were first grown to a late logarithmic phase with shaking and then left standing (without shaking) for 12–60h. The ratio of cells with polar localized CheZ-GFP in standing cultures was recorded at different times. After 24h, the number of cells with a polar localization pattern increased from 20 to 30% (Figures 1B,C). When cells were cultured without shaking for 60h, about 80% cells had polar localized CheZ-GFP (Figure 1C). *Azorhizobium caulinodans* with a GFP control vector showed a diffuse localization pattern under all conditions tested (Figure 1B).

**Figure 2:**
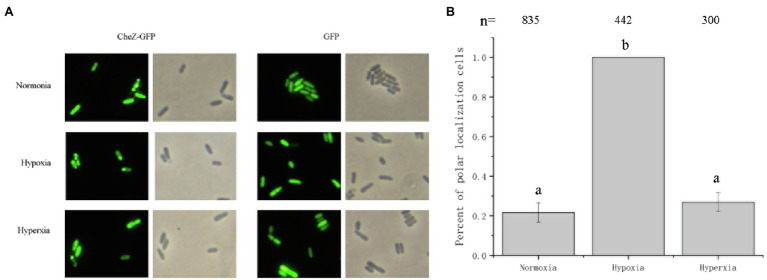
Localization of CheZ with different oxygen concentrations. **(A)** Representative images of localization of CheZ under normoxia, hypoxia, or hyperxia conditions after culturing for 12h with shaking. **(B)** Quantification of the ratio of cells with polar localization of CheZ under normoxia, hypoxia, or hyperxia conditions. The values are shown as the means±SDs from at least three independent experiments. The same letter above the bars indicates no significant difference at *p*<0.01, one-way ANOVA with Tukey’s multiple-comparison test.

The concentration of available oxygen becomes limited in cultures when cells are left standing for an extended period (Yuan et al., 2013). Thus, it was hypothesized that the increase in polar localization of CheZ without shaking may be induced by hypoxia. Environments with three kinds of oxygen concentrations were constructed using anerobic tubes to determine the localization pattern of CheZ. Under hypoxia conditions (Oxygen less than 4%) for 12h, around 100% cells showed polar localization (Figure 2). However, under hyperoxia conditions (Oxygen 33%), there were no obvious changes in CheZ localization compared with that under normoxia (Figure 2). These results indicate that oxygen limitation leads to the increase in polar localized CheZ.

### The Localization of CheZ Is Affected by the Energy Status of Cell

**Figure 3:**
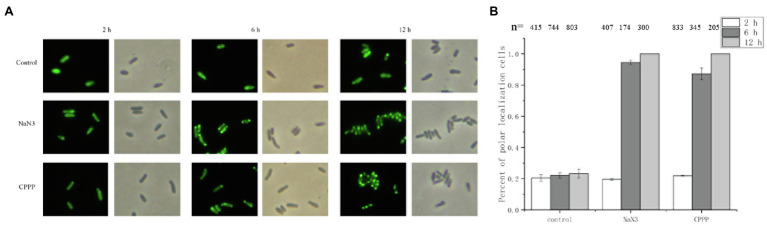
Localization of CheZ with electron transport chain inhibitors. **(A)** Representative images of CheZ localization in cells incubated with electron transport chain inhibitors sodium azide or CCCP, and with shaking for 12h. Cell cultures without inhibitor were used as control. **(B)** Quantification of cell ratios with polar localization of CheZ. Data are shown as means±SDs from three independent experiments.

Oxygen limitation could affect the energy status of the cells (Wessel et al., 2014). To determine whether the localization of CheZ is energy-related, two electron transport chain inhibitors, sodium azide and CCCP (Danylovych, 2016), were used. Sodium azide (0.2%) or CCCP (25μM) was added to cultures in the late logarithmic phase of growth. After incubating with shaking for 2h, there was no obvious difference between the control and inhibitor treatment in the localization pattern of CheZ (Figure 3). However, after 6h, the polar localization of CheZ increased to about 90% and reached 100% after 12h (Figure 3). These results indicate that the diffuse localization of CheZ relies on the energy status of the cell, and the cellular energy might be necessary to release CheZ from polar location.

### The C-Terminus and a Conserved Active Site D165 of CheZ Are Involved in the Energy-Related Polar Localization

**Figure 4:**
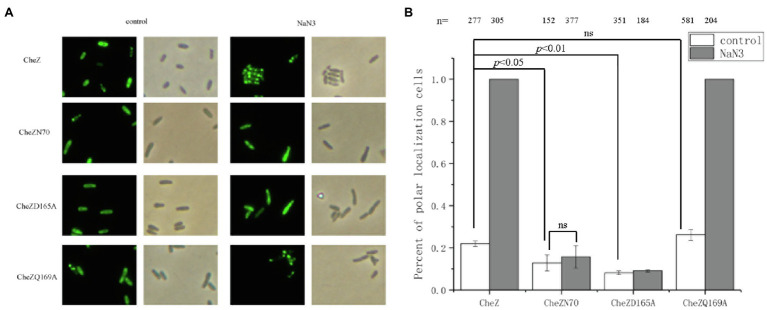
Role of the C-terminus of CheZ and phosphatase active sites on the localization of CheZ. **(A)** Images of cellular localization of the CheZN70, which the 70 residues at the N-terminus of CheZ was remained, and two CheZ substitution mutants CheZD165A and CheZQ169A. **(B)** Quantification of cell ratios with polar localization of CheZ, CheZN70, CheZD165A or CheZQ169A. Data are means and SDs from three independent experiments. The significant difference compared with control was analyzed by one-way ANOVA with Tukey’s test (*p*<0.01 and *p*<0.05).

Recently, we have mapped the region of CheZ for polar localization and found that the remaining of 70 residues at the N-terminus, CheZN70 (also termed as CheZΔ71-236), is sufficient for polar localization (Liu et al., 2020). To investigate whether the presence of N-terminal region is sufficient for the energy-related localization, the localization pattern of CheZN70 with or without sodium azide was recorded. After incubating with sodium azide for 12h, the CheZ localization pattern of CheZN70 remained unchanged as most cells had diffuse CheZ localization (Figure 4). These results suggest that the energy-related localization of CheZ may need its phosphatase activity.

Then we turned to test whether the energy-related localization of CheZ depends on its phosphatase activity. The conserved phosphatase active sites D165 and Q169 of CheZ in *A. caulinodans* ORS571 are important for its activity (Zhao et al., 2002; Silversmith et al., 2003; Lertsethtakarn and Ottemann, 2010; Liu et al., 2018b). We substituted these residues by alanine, respectively, and the resulting muteins CheZD165A and CheZQ169A were fused to GFP. When treated with sodium azide for 12h, the polar localization of CheZQ169A-GFP increased to 100%, which was similar as CheZ-GFP, while the localization pattern of CheZD165A-GFP in cells remained diffuse (Figure 4). These results indicate the conserved active site D165 is important for the energy-related localization; however, the conserved active site Q169 does not.

In addition, when some residues at the N-terminal helix of CheZ were deleted, such as CheZΔ2-31 and CheZΔ2-50, which reduced the polar localization of CheZ, almost 100% of cells with these truncated CheZ showed polar localization with the presence of sodium azide (data not shown). These results indicate that the C-terminus of CheZ and a conserved active site D165, but not N-terminal regions, are essential for the energy-associated localization.

### Role of Cytoplasmic Chemoreceptors Containing PAS Domains on the Localization of CheZ

**Figure 5:**
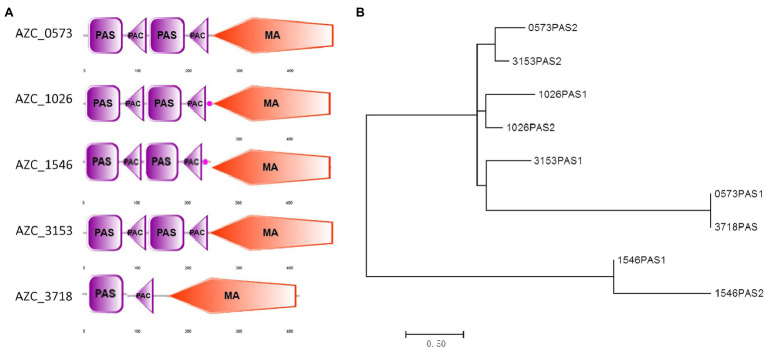
Analysis of soluble receptors in *A. caulinodans* ORS571. The domains in each receptor were showed on the schematic diagram **(A)**. The phylogenetic tree of PAS domains based on an alignment of amino acids sequences by MEGA7 **(B)**.

Changes in the electron transport system are a source of aerotaxis signals (Taylor et al., 1979; Zhulin et al., 1997a), and cytoplasmic chemoreceptors containing PAS domains can sense the status of redox and oxygen in cells (Zhulin et al., 1996, 1997b). Among *A. caulinodans* ORS571 genome, six cytoplasmic chemoreceptors were found, and five of them were closely phylogenetically related to *E. coli* Aer (Supplementary Figure S1). Analyzing sequences of the five chemoreceptors, all of them have PAS domain and the conserved methyl-accepting signal (MA) domain (Figure 5A). Four of them, including AZC_0573, AZC_1026, AZC_1546, and AZC_3153, contain two PAS domains, while AZC_3718 (also named as IcpB) only has one PAS domain (Figure 5A). After aligning PAS domains of these intracellular chemoreceptors, except two PAS domains in AZC_1546, others showed a close relationship with PAS domains in Aer in *E. coli*, and in AerC in *A. barasilense* (Figure 5B). Interestingly, two PAS domains from AZC_1026, two PAS domains from AZC_3153, and the PAS2 of AZC_0573 are close to each other on phylogenetic tree (Figure 5B), suggesting they may play redundant functions.

**Figure 6:**
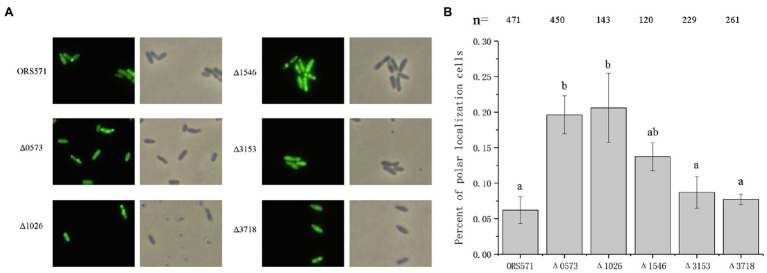
Effect of soluble receptor on the localization of CheZ. **(A)** Representative images of CheZ localization observed in five intracellular receptor single mutants (Δ*azc_0573*, Δ*azc_1026*, Δ*azc_1546*, Δ*azc_3153*, and Δ*azc_3718*). **(B)** Quantification of cells rations with polar localization of CheZ in different intracellular single receptor mutants. Values are means±SDs from at least three independent experiments. The same letter above the bars indicates no significant difference at *p*<0.05 by one-way ANOVA with Tukey’s multiple-comparison test.

The effect of these chemoreceptors on the localization of CheZ was further investigated. Deletion mutants of these chemoreceptors were constructed, and the CheZ-GFP fusion was introduced into each of them. The pattern of localization of CheZ in these mutants was compared with that in wild-type strain. When cells were cultured to the stationary phase, the ratio of cells showed polar localization of CheZs in Δ*azc_0573*, Δ*azc_1026*, and Δ*azc_1546* increased from 6% to around 20, 20, and 15%, respectively, while the localization pattern of CheZs in Δ*azc_3153* and Δ*azc_3718* remained unchanged (Figure 6). These results suggest that some intracellular chemoreceptors, AZC_0573, AZC_1026, and AZC_1546, participated in the regulation of CheZ localization.

### Free-Living Nitrogen Fixation Affects the Localization of CheZ

**Figure 7:**
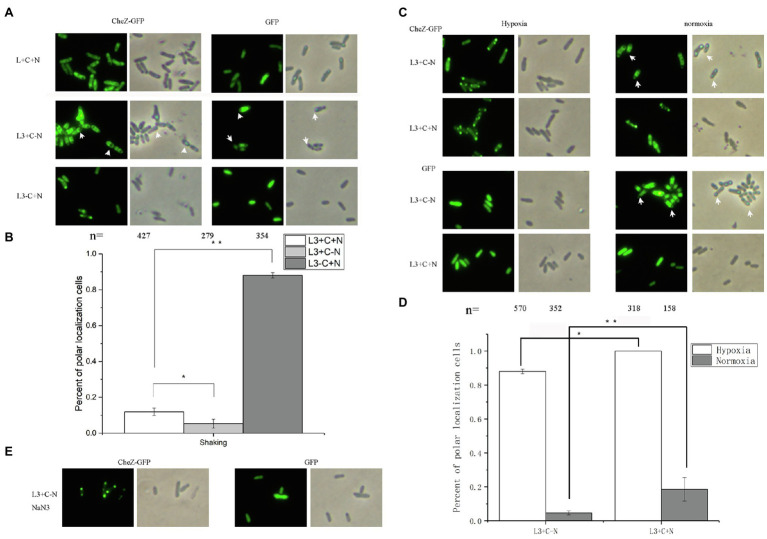
Localization of CheZ under nitrogen limiting and free-living nitrogen fixation conditions. **(A,B)** Images and quantified data of cells of *A. caulinodans* ORS571 under nitrogen limiting conditions (shaking with normoxia and without nitrogen source). **(C,D)** Representative images and quantification of cells with polar localization of CheZ under hypoxia conditions of nitrogen fixation (hypoxia without nitrogen source) or non-nitrogen fixation (hypoxia with nitrogen source; **C** left panel and **D**). Then the cells are transferred from under hypoxia conditions of nitrogen fixation conditions (hypoxia without nitrogen source) or non-nitrogen fixation (hypoxia with nitrogen source) to normoxia conditions (**C** right panel and **D**). The effect of nitrogen limiting on localization of CheZ depends on the energy status of cells **(E)**. The carbon limiting condition was used as a control. Data are means±SDs from at least three independent experiments. Asterisks (^*^p<0.05, ^**^p<0.01) show a significant difference between two samples according to *t*-test.

Nitrogenase activity can only be detected at low concentration of oxygen (less than 4%) and nitrogen limiting conditions (Desnoues et al., 2003), suggesting that the hypoxia induced polar localization of CheZ may be related to nitrogen fixation. Before testing the CheZ localization under nitrogen fixation condition (hypoxia and nitrogen-limitation), we first tested the effect of nitrogen limiting on the CheZ localization. Under nitrogen limiting conditions without shaking, nitrogen-fixing rhizobacteria can move toward suitable regions with microaerobic for nitrogen fixation (Greer-Phillips et al., 2004), which was called aerotaxis. To avoid the effect of aerotaxis, we compared the localization pattern of CheZ in the presence or absence of ammonium with shaking. Compared to the group with nitrogen source (L3+C+N), the nitrogen limitation (L3+C-N) decreased the ratio of CheZ polar localization from 10 to 5% of cells showed (Figures 7A,B). For some bacteria, under nitrogen limiting conditions and excessive carbon source, they could accumulate glycogen, polyhydroxyalkanoates, or other substances to response to harsh environments (Blaby et al., 2013; Mozejko-Ciesielska et al., 2018; Lai et al., 2019). Interestingly, under nitrogen limitation, CheZ-GFP proteins in many cells are squeezed from both ends and converge in the middle of cells (Figure 7A), which is different from common diffuse localization pattern, suggesting *A. caulinodans* may also produce glycogen-like molecules affecting the distribution of CheZ-GFP. The cells cultured with carbon starvation (L3-C+N) were used as a control group, and CheZ-GFP in about 90% cells locates to cell poles, which is similar as the localization pattern in the presence of sodium azide (Figures 7A,B).

Then, we tested the role of free-living nitrogen fixation on CheZ localization. Under nitrogen fixation condition (hypoxia and absence of ammonium), about 90% cells showed polar localization of CheZ (Figures 7C,D). However, 100% cells showed polar localization of CheZ under hypoxia with nitrogen source (Figures 7C,D). Because no CheZ-GFP is squeezed in the middle of cells under nitrogen fixation conditions (Figure 7C), the role of nitrogen limitation can be excluded. Interestingly, when we transferred cells from hypoxia conditions of nitrogen fixation (hypoxia without nitrogen source) or non-nitrogen fixation (hypoxia with nitrogen source) to normoxia conditions, the ratio of cells with CheZ polar localization of them both decreased significantly (Figures 7C,D). With the decrease in CheZ polar localization, the squeezed localization pattern of CheZ appeared again when cells transferred from nitrogen fixation condition to nomoxia condition (Figure 7C), suggesting without nitrogen fixation, cells begin to suffer nitrogen limitation again. These results suggest the free-living nitrogen fixation of bacteria might partially release the degree of CheZ polar localization. When sodium azide was added into cell cultures under nitrogen fixation conditions, 100% cells showed a polar localization of CheZ (Figure 7E), supporting the hypothesis that the decreased localization pattern caused by nitrogen fixation is also energy dependent.

## Discussion

Eukaryotic cells have membrane-bound organelles, which can increase the efficiency of interaction between components and reduce cross interference. Prokaryotic cells do not have membrane-bound organelles, thus the subcellular organization of proteins is important for the competitiveness and survival of them. Polar localization is closely related to the lifecycle of bacteria (Gestwicki et al., 2000). For example, *Vibrio parahaemolyticus* has two different cell types (swimmer cell or swarmer cell), and swarmer cells showed a lateral localization of chemotaxis proteins, which is different from that in swimmer cells (Heering and Ringgaard, 2016). In addition, subcellular localization is linked with the phosphorylated level of proteins. In *Pseudomonas aeruginosa*, a hybrid response regulator-diguanylate cyclase, WspR, which is involved in the response to growth on surfaces, can form subcellular clusters when it is phosphorylated (Guvener and Harwood, 2007; Huangyutitham et al., 2013). Furthermore, the polar localization of chemotaxis proteins improves the cooperative interactions between them, which enables bacteria sense nanomolar concentrations of signal molecules over a wide range (Greenfield et al., 2009).

We previously identified the residues and domains of CheZ, which are important for the cellular localization of CheZ (Liu et al., 2020). In this study, we further found that the location of CheZ can be regulated by hypoxia. Through disrupting the respiratory chain of *A. caulinodans* ORS571 with sodium azide or CCCP, we confirmed that the localization pattern of CheZ is energy related. In *V. cholerae*, the localization of three sets of chemotaxis signaling proteins (system I, II, and III) was determined (Hiremath et al., 2015; Ringgaard et al., 2015). All of them could form polar clusters, and the localization patterns of two non-chemotaxis systems (system I and III) are also energy-related, which can be induced by starvation or hypoxia (Hiremath et al., 2015; Ringgaard et al., 2015). Different from them, CheZ is a chemotactic protein which plays important roles in the chemotaxis signal transduction pathway of *A. caulinodans* ORS571, and the substitution of the conserved phosphatase active sites with alanine could abolish the effect of CheZ on chemotaxis (Liu et al., 2018b). The first 70 residues at N-terminus of CheZ are sufficient for its polar localization (Liu et al., 2020). In this study, we determined CheZ localizes to the poles upon inhibiting the electron transport chain of *A. caulinodans*, which requires the C-terminus of CheZ and one of the conserved phosphatase active site, D165 but not Q169. These results are consistent with our previous observation that the substitution mutant of CheZ (CheZD165A) showed a low level of localization, while the localization pattern of CheZQ169A was similar to wild-type CheZ (Liu et al., 2020). Recently, we identified that a novel motif AXXF(Y)Q, which is adjacent to the phosphatase active sites, controls the polar localization of CheZ (Liu et al., 2020), and we presumed the role of D165 of CheZ on localization might be achieved by interacting with the motif.

Aerotaxis or energy taxis is a common behavior of soil bacterial, which guides bacteria toward best niche for survival. The energy taxis response has been well studied in *E. coli* and *A. basilense*, which can sense the changes in the electron transport chain trough cytoplasmic receptors, such as Aer and AerC (Alexandre et al., 2000; Greer-Phillips et al., 2003). It was reported that the localization of AerC correlates with the energy status of cells in *A. brasilense*, which is sensed by PAS domain (Xie et al., 2010). In *A. caulnodans* ORS571, five Aer homologs containing PAS domains were identified; however, we determined only three of them were involved in the regulation of CheZ localization. Both AZC_3153 and AZC_3718 are highly conserved to one another, suggesting their role may be redundant. The regulatory mechanism of CheZ localization by five intracellular chemoreceptors needs to be further investigated.

No matter under carbon or nitrogen limiting conditions, *A. caulinodans* ORS571 cells cannot grow (Liu et al., 2019), but showed opposite localization patterns of CheZ (polar or diffuse). Under nitrogen limitation and excess carbon source conditions, cells can form glycogen, polyhydroxyalkanoates, or other substances to response to harsh environments (Blaby et al., 2013; Mozejko-Ciesielska et al., 2018; Lai et al., 2019). Under the condition with carbon source and without a nitrogen source, the glycogen-like substances formed by *A. caulinodans* ORS571 cells may squeeze the GFP-fused protein aside. When sodium azide was added, there was no glycogen-like substances formed and cells showed 100% polar localization of CheZ, indicating the diffuse localization pattern of CheZ might be dependent on the energy status in cells forming glycogen-like substances.

What are the possible physiological roles of the hypoxia-induced polar localization? In the lifecycle of *A. caulinodans* ORS571, hypoxia is essential for nitrogen fixation, no matter in free-living states or inside nodules of *S. rostrata*. In soil environments without nitrogen source, *A. caulinodans* ORS571 moves toward low oxygenated area to fix atmospheric nitrogen. The results in this work raise the possibility that, in *A. caulinodans* ORS571, CheZ plays roles in the nitrogen fixation under hypoxia. According to the reports that a free-living rhizospheric bacterium *Pseudomonas stutzeri* A1501 can form biofilm to fix nitrogen under aerobic conditions (Wang et al., 2017), and CheZ in *A. caulinodans* ORS571 inhibits the formation of biofilm and EPS (Liu et al., 2018b), whether the roles of CheZ on EPS production and biofilm formation can be affected by hypoxia should be studied in the future.

In conclusion, polar localization of CheZ in *A. caulinodans* ORS571 was induced by hypoxia or energy status in cells. This is the first study to report the relationship between hypoxia and “real” chemotactic proteins, and the relationship between hypoxia, free-living nitrogen fixation, localization of CheZ, and the energy sensing chemoreceptors sheds new light on the functional regulation of chemotactic proteins.

## Data Availability Statement

The raw data supporting the conclusions of this article will be made available by the authors, without undue reservation.

## Author Contributions

XL and ZX conceived and designed the experiments, analyzed the data, prepared the figures and tables, and wrote the manuscript. XL, YL, YW, and DW carried out the experiments. KJ helped with the improvement and revision of the manuscript. All authors contributed to the article and approved the submitted version.

## Funding

This work was financed by the National Natural Science Foundation of China (31870020), the Strategic Priority Research Program of the Chinese Academy of Sciences (XDA23050102), and National Key Research and Development Program (2019YFD1002702) to ZX.

## Conflict of Interest

The authors declare that the research was conducted in the absence of any commercial or financial relationships that could be construed as a potential conflict of interest.

## Publisher’s Note

All claims expressed in this article are solely those of the authors and do not necessarily represent those of their affiliated organizations, or those of the publisher, the editors and the reviewers. Any product that may be evaluated in this article, or claim that may be made by its manufacturer, is not guaranteed or endorsed by the publisher.

## Footnotes

^1^ https://mistdb.com/
