# Supplementary material for: The Hypoxia-Associated Localization of Chemotaxis Protein CheZ in Azorhizorbium caulinodans
Source: Front Microbiol. 2021 Oct 15;12:731419. doi: 10.3389/fmicb.2021.731419 (PMC8563088; doi:10.3389/fmicb.2021.731419)
Supplement: Supplementary file 1 [file Data_Sheet_1.docx]

***Supplementary Material***



Figure S1. The phylogenetic tree of seven receptors from *A. caulinodans* ORS571 and Aer from *E. coli*. The phylogenetic tree was based on an alignment of amino acids sequences of these receptors using maximum likelihood method.
